# Tracking the morphological evolution of neuronal dendrites by first-passage analysis

**DOI:** 10.1016/j.bpj.2025.11.005

**Published:** 2025-11-07

**Authors:** Fabian H. Kreten, Barbara A. Niemeyer, Ludger Santen, Reza Shaebani

**Affiliations:** 1 Department of Theoretical Physics, Saarland University, Saarbrücken, Germany; 2 Center for Biophysics, Saarland University, Saarbrücken, Germany; 3 Department of Molecular Biophysics, Center for Integrative Physiology and Molecular Medicine, School of Medicine, Saarland University, Homburg, Germany

## Abstract

A high degree of structural complexity arises in dynamic neuronal dendrites due to extensive branching patterns and diverse spine morphologies, which enable the nervous system to adjust function, construct complex input pathways, and thereby enhance the computational power of the system. Recognition of pathological changes due to neurodegenerative disorders is of crucial importance due to the determinant role of dendrite morphology in the functionality of the nervous system. Nevertheless, direct noninvasive measurements to collect adequate structural data in a reasonable time are currently not feasible. Here, we present a stochastic coarse-grained framework based on first-passage analysis to infer key dendritic morphological features affected by neurodegenerative diseases—including the density and size of spines, the extent of the tree, and the segmental increase of dendrite shaft diameter toward the soma—from the statistical characteristics of a measurable temporary signal generated by tracers that have diffusively passed through the complex dendritic structure. Thus, our theoretical approach can provide a noninvasive route to link dendritic morphology with possible accessible readouts in neurodegenerative disease monitoring. As a prospective application, we discuss how externally detectable signals could be realized in practice, suggesting potential pathways toward experimental implementation.

## Significance

Neurodegenerative disorders significantly change the morphology of neuronal dendrites. Hence, monitoring the morphological evolution is crucial to diagnose and predict diseases and to monitor success of potential treatments. However, it is currently not feasible to collect adequate structural data in a reasonable time by direct noninvasive measurement methods. To address this gap, we develop a stochastic theoretical approach to extract essential morphological information of evolving dendrites from the statistical properties of externally measurable signals. Although the primary contribution of this study is to establish the theoretical foundation for this connection, we also outline how a detectable signal might be experimentally realized, pointing toward future noninvasive strategies for monitoring dendritic pathology.

## Introduction

The elaborate branching morphology of neuronal dendrites in advanced nervous systems allows a single neuron to interact simultaneously with multiple other neurons through their axon terminals, leading to a complex network of signaling pathways (1,2). The diverse functions of dendritic trees are reflected in the broad variation of their architecture in different neuronal types and regions. The complex behavior of higher animals has also been attributed to the presence of small membranous protrusions called dendritic spines (3,4,5,6). Functional synapses, as the basic computational units of signal transmission, consist of the presynaptic release site and dendritic protrusions, harboring the signal recognition and transmission units. Spines play a vital role in neural functions such as cognition, memory, and learning (7,8,9,10,11), serving as the recipients of excitatory and inhibitory inputs in the mammalian brain and undergoing dynamic structural changes regulated by neuronal activity (12,13). The morphology of spines plays a crucial role as, for example, the shape and size of spine head and neck determine the number of postsynaptic receptors and the generated synaptic current (8) and control the electrical and biochemical isolation of the spine from the dendrite shaft (14,15).

Aging and several neurodegenerative diseases—for example, fragile X and Down syndromes, Alzheimer disease, schizophrenia, and autism spectrum disorders—significantly influence the function of the nervous system by altering the morphology of dendrites (16,17,18,19,20). These alterations occur in the overall extent of dendritic trees, the population of branches, the thickness and curvature of dendrite shafts, and the density, morphology, and spatial distribution of spines (21,22,23,24,25,26,27,28,29,30,31,32,33,34). On the other hand, reversal of morphological changes upon treatment has also been reported (35,36). Despite the crucial importance of monitoring the structural evolution of dendritic trees and spines to diagnose and predict neurodegenerative diseases and to monitor success of potential treatments, noninvasive imaging of neuronal dendrites is currently infeasible. It is even highly challenging to collect statistically adequate structural information from direct invasive imaging due to technical limitations: although image analysis techniques for 3D reconstruction of dendrites have been improved in recent years (37,38), a high resolution image can be achieved by electron microscopy, which is a very laborious technique and practically inappropriate for spatially large-scale investigations. Nevertheless, there exist powerful noninvasive techniques that allow real-time tracking of brain activities, ranging from electro- and magneto-encephalography for electric and magnetic field detection (39,40) to nuclear magnetic resonance spectroscopy, positron emission tomography, and magnetic resonance imaging (MRI) for measuring the concentration of (neuro-)chemicals (41,42,43,44).

In the absence of a direct, efficient method for acutely unraveling the microscopic morphology of spines and dendrites, we propose a complementary strategy: using statistical analysis of externally detectable signals generated by a large population of neurons to indirectly infer essential structural features. In this theoretical work, we develop a stochastic coarse-grained framework based on first-passage analysis of tracer particles through the dendrite structure. We demonstrate how key morphological characteristics of dendrites, including spine density and size, dendritic extent, and tapering toward the soma, are encoded in the statistical properties of evolving signals. Although the generation of such externally measurable signals remains speculative, our framework illustrates how theoretical insights could be coupled with experiments to enable noninvasive monitoring of dendritic morphology. In the discussion section, we outline possible experimental avenues for realizing such detectable signals, with the long-term goal of systematic monitoring of neurodegenerative disease or treatment progression for individual patients.

## Materials and methods

### Coarse-grained dendrite model

**Figure 1:**
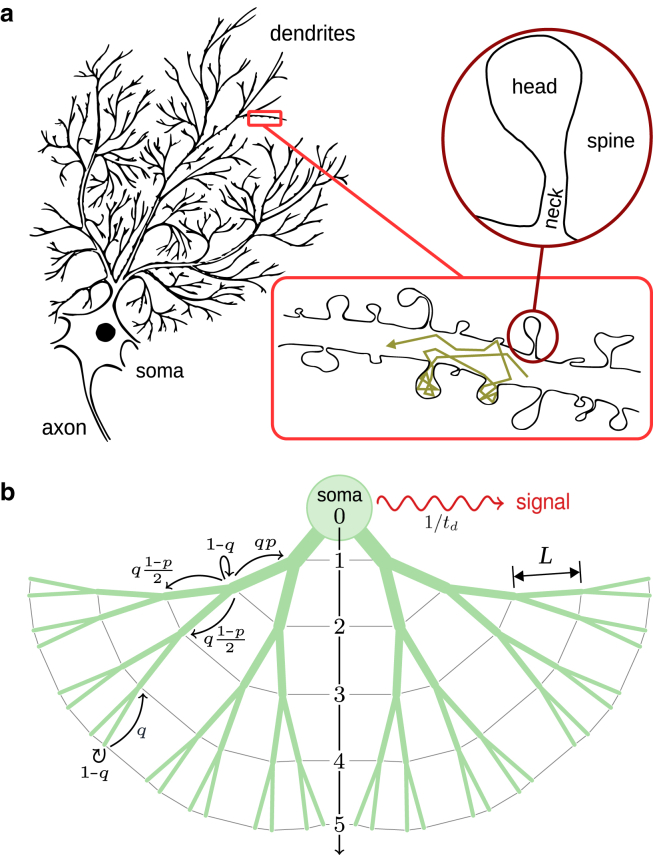
Morphology of neuronal dendrites. (*a*) Schematic drawing of a neuron. Insets: (*lower*) a section of a typical dendritic channel. A sample path of a particle is shown; (*upper*) structure of a mushroom-like spine. (*b*) Sketch of our binary tree model. A tree structure with depth *n* = 5 is depicted as an example. The possible choices at junctions or dead ends are shown with arrows. The coarse-grained probability to reach a neighboring intersect is denoted with *q*, the topological bias *p* represents the segmental increase of dendrite diameter toward the soma, and 1/*t*_*d*_ denotes the pulse emission rate.

We model the structure of dendrites by adopting a mesoscopic perspective and considering a regularly branched tree with *n* generations of junctions. The average distance between adjacent nodes is denoted by *L*; thus, the tree has a linear extent *nL*. Importantly, although here we restrict ourselves to symmetric trees, our results remain valid under realistic levels of global variation in structural parameters or local irregularities across the tree architecture (45,46). By coarse-graining the stochastic transport within dendritic shafts and spines, we study the dynamics of noninteracting particles hopping between the nodes of the tree (see (45); Fig. 1). Using the resolution of measurement or observation Δ*t* as the time unit, we resolve the dynamics at the coarse-grained timescale Δ*t*. Each particle, drawn from an initial reservoir containing N particles, enters the tree at a random node indexed by its depth *i*∈[0,*n*], where *i* = 0 corresponds to the soma and *i* = *n* to the dead ends. The time of entry is randomly sampled from a geometric distribution with mean *t*_*e*_ (measured in units of Δ*t*), consistent with the goal of monitoring transport behavior over a brain region rather than focusing on individual particles. Thus, at each time step, each reservoir particle enters the tree with probability 1/*t*_*e*_. Assuming a uniform probability of entry site across the tree, the entry probability increases exponentially with node depth in a binary tree, yielding a distribution 2^*i*−1^/(2^*n*^-1) for entering at an entry level *i* (*i* ≥ 1). Thus, the joint probability that a particle enters the dendrite binary tree at time *t* on the level *i* is given by $E_{i}\left(t\right)=\frac{1}{t_{e}}{\left(1-\frac{1}{t_{e}}\right)}^{t}\frac{2^{i-1}}{2^{n}-1}$.

At each time step, a particle either hops to a neighboring node with probability *q*, or remains at its current position with probability 1 − *q*. The waiting probability accounts for both stochastic trapping inside dendritic spines and the diffusive delay within the dendrite channel. We assume that the residence probability inside these biochemical cages is depth independent since the spine number density along the dendrite is known to rapidly saturate after a short distance from the soma (37,47). Denoting the mean escape time from a junction to a neighboring one with *τ*, Δ*t* is related to the hopping probability via Δ*t* = *qτ*. To model the directional preference in tracer particle motion toward the soma or dead ends, we introduce a topological bias parameter *p*: transitions toward the soma and toward the dead ends occur with probabilities *p* and 1 − *p*, respectively. The boundary conditions at the outer boundaries, including the dead ends and the soma, are chosen as follows: terminal branches are treated as reflective; that is, a particle that reaches a dead-end leaves it and returns to the previous bifurcation with probability *q*, which effectively accounts for both the mean travel time back to the bifurcation and possible delays due to entrapment in spines along the terminating branch. The soma, by contrast, is treated as an absorbing boundary, consistent with our previous work on first-passage times to the soma (45). Once a particle reaches the soma, it emits a transient pulse after a randomly sampled delay, referred to as the emission time, drawn from a geometric distribution with mean *t*_*d*_ (measured in units of Δ*t*). We assume that the particle emits a single pulse with probability $\frac{1}{t_{d}}$, after which it is removed from the system. Physically, this can represent either a tracer that cannot leave the soma and reacts irreversibly after a random delay, or a tracer that becomes activated upon soma contact, producing a measurable signal independent of its precise position. Although we assume absorption at the soma and a specific form of signal generation for illustration, the boundary conditions at the soma and dead ends and the initial conditions of entering the tree in our framework are flexible and can be adapted to match any relevant experimental setup.

The probability *F*_*i*_(*t*) for a particle being at depth level *i* at time *t* can be obtained by solving the following set of coupled master equations together with the initial condition of an empty dendritic tree, *F*_*i*_(0) = 0 for all *i*∈ {0,1, …,*n*}. The evolution of the system from time *t* to *t*+ 1 is governed by the following (48):

(1) $$\left\{ \begin{array}{cc} F_{0}\left(t+1\right) & =\left(1-1/t_{d}\right)F_{0}\left(t\right)+qpF_{1}\left(t\right)\text{,}\\ F_{1}\left(t+1\right)\; & =\left(1-q\right)F_{1}\left(t\right)+qpF_{2}\left(t\right)+E_{1}\left(t\right)\text{,}\\ \vdots & \\ F_{i}\left(t+1\right)\; & =q\left(1-p\right)F_{i-1}\left(t\right)+\left(1-q\right)F_{i}\left(t\right)+qpF_{i+1}\left(t\right)+E_{i}\left(t\right)\text{,}\\ \vdots & \\ F_{n-1}\left(t+1\right) & =q\left(1-p\right)F_{n-2}\left(t\right)+\left(1-q\right)F_{n-1}\left(t\right)+qF_{n}\left(t\right)+E_{n-1}\left(t\right)\text{,}\\ F_{n}\left(t+1\right)\; & =q\left(1-p\right)F_{n-1}\left(t\right)+\left(1-q\right)F_{n}\left(t\right)+E_{n}\left(t\right)\text{.} \end{array}\right.$$

By solving the above equations, the particle dynamics and related quantities such as the first-passage time distribution can be extracted. The resulting dynamics of individual particles can be described in general by stochastic two-state models (49,50). In particular, we previously derived the mean first-passage time (MFPT) of being absorbed in the soma (though for the initial condition of only entering from the dead ends) in terms of the structural parameters {*n*,*q*,*p*} by treating the soma as an absorbing boundary (45,46). Importantly, we verified that the analytical predictions remain valid for realistic degrees of structural fluctuations (e.g., diversity in the extent of tree along different directions, disorder in the local branching patterns, etc.). Although the high sensitivity of the MFPT to the structural characteristics of dendrites is promising, MFPT is not a directly measurable quantity in dendrites. To realize the practical potential of our approach in technology and medicine, here, we extend our proposed formalism and consider the subsequent steps after the particles reach the soma. We assume that each particle emits a temporary pulse after reaching the soma. The accumulation of the pulses generated in many neurons results in an evolving overall signal intensity *I*(*t*) that can be detected externally. *I*(*t*) is related to the probability *F*_*i*_(*t*) through $I\left(t+1\right)=\frac{1}{t_{d}}F_{0}\left(t\right)$.

The signal *I*(*t*) contains the information of entering, first-passage, and emission times. It indeed reduces to the first-passage time distribution (though shifted by two time steps) in the limit of instantaneous entering and pulse emission (i.e., *t*_*e*_ = *t*_*d*_ = 1). For the general case of *t*_*e*_,*t*_*d*_ > 1—where *I*(*t*) deviates from the first-passage time distribution—we demonstrate how the statistical characteristics of *I*(*t*) can be linked to the morphological properties {*n*,*q*,*p*} of the dendrite structure. We note that the location of signal generation is in principle arbitrary. Here, we choose the soma as the signal generation point for simplicity—since this choice reduces the problem to an effective 1D lattice—but the approach is extendable to alternative scenarios with different signal generation locations.

The details of calibration of the model parameter *q*, clarification of the required time resolution of measurements, and estimation of the applicability range of our proposed technique are presented in the Supporting Material.

## Results

### Mapping to real dendrite structures

We first verify the applicability of our mesoscopic approach by mapping the coarse-grained model parameters to the morphological characteristics of real dendrite structures. The depth parameter *n* and node-to-node distance *L* are directly mapped to the extent of the dendritic tree, which primarily depends on the nervous system and neuronal region and type. For instance, cerebellar Purkinje cells of guinea pigs extend up to 200*μ*m from the soma and have ∼ 450 dendritic terminals (51). This corresponds to nearly *n* = 10 generations of junctions that branch out around every *L* = 20*μ*m.

**Figure 2:**
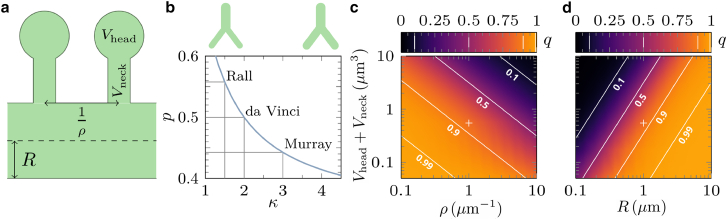
Calibration of the mesoscopic model parameters. (*a*) Sketch of a section of the dendrite channel. (*b*) Bias probability *p* in terms of the allometric exponent *κ*. The corresponding points for a couple of known structures are marked. The insets show schematic drawings of channel diameters at different *κ* regimes. (*c* and *d*) Moving probability *q* in the (*V*_head_+*V*_neck_, *ρ*) and (*V*_head_+*V*_neck_, *R*) planes for the maximum possible time step $\Delta t=\frac{L^{2}}{2D}$. Other parameters are (*c*) *R* = 1*μ*m and (*d*) *ρ* = 1*μ*m^−1^. The solid white lines represent isolines of constant *q* with the indicated values. The crosses mark the set of parameter values (*V*_head_+*V*_neck_ = 0.55*μ*m^3^, *ρ* = 1*μ*m^−1^, *R* = 1*μ*m) for a typical healthy dendrite as a reference for comparison.

To map the model parameters *p* and *q* to the structure of real dendrites, we consider the diffusive dynamics of tracer particles along a dendritic tree with protrusions as depicted in Fig. 2 *a*. The bias *p* in the direction of motion arises from geometrical asymmetries such as tapering of the channel cross section as well as branching at the junctions into *z* − 1 children (*z* being the number of branches at each junction). The directional bias can be approximated as follows (48):

(2) $$p=\frac{A_{p}}{A_{p}+\left(z-1\right)\;A_{c}}\text{,}$$

where *A*_*p*_ and *A*_*c*_ denote the cross-sectional areas of the parent and child branches, respectively. The areas can be extracted through the allometric relation $d_{p}^{\;\kappa }=\sum_{i=1}^{z-1}d_{c_{i}}^{\;\kappa }$ between the diameters of the parent and child branches at the junction. Here, *d*_*p*_ and $d_{c_{i}}$ denote the parent and *i*-th child branch, respectively, and *κ* is the allometric exponent (52). Empirical and theoretical studies suggest representative values of the allometric exponent, with $\kappa =\frac{3}{2}$ for dendrites of motor neurons (Rall, 1959) (53), *κ* = 2 for botanical trees (da Vinci’s exponent (54)), and *κ* = 3 for vascular and pulmonary networks (Murray’s exponent(55)). Other exponents in neuronal context were found to be *κ* = 2 ($p=\frac{1}{2}$) for Purkinje cells, *κ* = 2.5 (*p* = 0.47) for peripheral nervous system neurons, and *κ* = 3 (*p* = 0.44) for axons (56). Using the allometric relation, Eq. (2) results in

(3) $$p=\frac{1}{1+{\left(z-1\right)}^{1-\frac{2}{\kappa }}}\text{.}$$

Assuming bifurcations (*z* = 3) and symmetric child branches yields, for example, estimated values of *p*≃ 0.56, 0.5, and 0.44, for $\kappa =\frac{3}{2}$, 2, and 3, respectively; see Fig. 2 *b*. For general channel geometries and driving forces, the bias parameter can be obtained by solving a Fick-Jacobs-like equation (48). Fig. 2 *b* illustrates that larger values of *p* correspond to a more pronounced thickening of the channels toward the soma.

To calibrate the probability of motion *q*, we equate the mean time to leave one node in the coarse-grained discrete time model with the mean travel time from the current junction to any of the neighboring ones in the presence of spines. We consider a symmetric branch at which the child channels are connected with equal radii and without leaving a void space. The entrapment of particles inside spines leads to an effective diffusion constant $D_{\text{eff}}=D\;\frac{V_{\text{channel}}}{V_{\text{channel}}+V_{\text{spines}}}$ for the diffusive dynamics inside the channel, where *V*_channel_ is the volume of the channel segment, *V*_spines_ is the total volume of spines along it, and *D* is the diffusion constant in the absence of spines (57). We obtain the following expressions for the moving probability (see Supporting Material for details):

(4) $$q=\Delta t\frac{2D_{\text{eff}}}{L^{2}}=\Delta t\frac{2D}{L^{2}}\frac{1}{1+\frac{\rho \left(V_{\text{head}}+V_{\text{neck}}\right)}{\pi R^{2}}}\text{,}$$

with Δ*t* being the time resolution of measurements, *V*_neck_ and *V*_head_ the spine neck and head volumes, *ρ* the spine density per length unit for regularly spaced spines along the channel, and *R* the radius of the channel segment. The above equation imposes no explicit bound on *q*; however, both *p* and *q* parameters are indeed restricted due to the limited biologically relevant ranges of the structural parameters. For example, *V*_head_+*V*_neck_≃ 0.5*μ*m^3^ and *ρ*≃ 1*μ*m^−1^ represent typical structural parameter values for a healthy dendrite (58). We also note that the diffusion constant depends on the particle size. Some typical values are as follows: *D*∼ 20 (green fluorescent protein (GFP) variants inside spines), ∼ 100 (large Ca^2+^ ions inside spines), ∼ 37 (photo activatable GFP, paGFP, inside dendrite channels), and ∼ 23.5*μ*m^2^/s (enhanced GFP, eGFP, inside the nucleus) (10,59,60). Using these values, we obtain *t* ≃ 0.8, 0.5, and 0.2 s for the escape time of eGFP, paGFP, and Ca^2+^ from spines and *t*≃ 8.5, 5.4, and 2.0 s for their travel time from one junction to the next one in a dendritic tree similar to that of cerebellar Purkinje cells but in the absence of spines. The behavior of *q* versus the structural parameters of dendrites is shown in Fig. 2 *c* and *d*. It can be seen that *q* varies monotonically with the structural parameters within their biologically relevant ranges, which allows for mapping of the morphological characteristics of real dendrite structures to the coarse-grained model parameters.

### Signal processing

To establish a direct link between the dendrite morphology and the statistical characteristics of the detectable signal, our next step is to demonstrate how the coarse-grained model parameters influence the time evolution of the overall signal. To compute the signal *I*(*t*), we perform Monte Carlo simulations with an ensemble of $N=10^{6}$ realizations of stochastic entry, first-passage, and emission times, as described in the “coarse-grained dendrite model” section. When a tracer reaches the soma, its stochastic emission time is sampled from a geometric distribution, and the tracer is added to the soma reservoir. Once the emission time is reached, the tracer generates an instantaneous pulse and is removed from the soma reservoir. Fig. S3 illustrates the evolution of the number of accumulated tracers at the soma for different dendritic morphologies and parameter values. The signal *I*(*t*) at a given time point *t* is computed as the number of instantaneous pulses emitted at that time (i.e., the number of tracers eliminated from the soma reservoir at time *t*), normalized by N. In the results shown, each emission is modeled as an instantaneous pulse of one time step duration. More generally, the framework allows for a stochastic emission period, during which a tracer contributes to *I*(*t*) across multiple time steps before being removed. We have recently shown that in general the detected signal *I*(*t*) from branched structures develops a peak followed by an exponential decay at long times (48). The location and height of the peak and the slope of the tail depend on the coarse-grained model parameters and the timescales *t*_*e*_ and *t*_*d*_. For small values of *t*_*e*_ and *t*_*d*_, the signal intensity is nearly equivalent to the first-passage time distribution of passing through the dendritic tree to reach the soma. Note that the signal intensity is invariant under the swapping of *t*_*e*_ and *t*_*d*_, and the asymptotic behavior is governed by the longest timescale. Overall, a faster arrival in the soma and/or a faster emission of the signal is associated with an earlier and higher peak and a steeper tail of *I*(*t*).

To develop a more quantitative understanding of how key model parameters govern the signal intensity evolution, we vary *n*, *q*, and *p* over the biologically relevant ranges and calculate various statistical characteristic of *I*(*t*). Of particular interest is the behavior of the logarithm of the median, *log*_10_(*Q*_1/2_). Our previous results revealed that the median of the signal intensity varies monotonically in terms of the structural parameters, even for large values of *t*_*e*_ and *t*_*d*_ (48). A similar behavior can be observed for the mean or maximum of *I*(*t*). Thus, measuring the median of the signal intensity (or any other quantity in this category) identifies an isosurface in the (*n*,*q*,*p*) space—defined as an admissible set of {*n*,*q*,*p*} parameter combinations along which *log*_10_(*Q*_1/2_) (or any other statistical characteristic of *I*(*t*)) remains constant. This is, however, insufficient to uniquely determine these parameters. For a unique determination of the parameters {*n*,*q*,*p*}, additional statistical characteristics of *I*(*t*) whose isosurfaces behave differently from the median ones need to be extracted. We tested several quantities, among them the variance, skewness, etc. We identified a second category of shape quantities that describe the dispersion of *I*(*t*). The isosurfaces of this category of quantities behave differently from the median’s ones but not significantly from each other. These two independent characteristics of *I*(*t*) allow for identifying at least a 1D subset in the (*n*,*q*,*p*) space. As a representative of the second category, we choose to work with the relative interquartile range Δ*Q*_*r*_, which is a measure of the statistical dispersion of *I*(*t*) and has a smooth behavior upon varying the structural parameters. It is defined as $\Delta Q_{r}=\frac{Q_{3/4}-Q_{1/4}}{Q_{3/4}+Q_{1/4}}$, with *Q*_*i*/4_ being the *i*th quartile (for example, the second quartile *Q*_2/4_≡*Q*_1/2_ corresponds to the median). We previously verified that the isolines of Δ*Q*_*r*_ and *log*_10_(*Q*_1/2_)—obtained by taking two-dimensional slices of the isosurfaces of constant *log*_10_(*Q*_1/2_) or Δ*Q*_*r*_ for a fixed value of *n*, *q*, or *p* parameter, i.e., the intersection of the isosurfaces with a constant *n*, *q*, or *p* plane—have distinctly different orientations at small *t*_*e*_ and *t*_*d*_ (48). In this regime, each pair of isolines for a measured set of *log*_10_(*Q*_1/2_) and Δ*Q*_*r*_ intersect at a single point. However, at longer *t*_*e*_ and *t*_*d*_ timescales, the isolines of Δ*Q*_*r*_ may exhibit a nonmonotonic behavior within the relevant range of the structural parameters or become nearly parallel to the isolines of *log*_10_(*Q*_1/2_). As a result, each pair of isolines for a measured set of *log*_10_(*Q*_1/2_) and Δ*Q*_*r*_ may have several intersections, meaning that {*n*,*q*,*p*} parameters cannot be uniquely determined. We note that identifying the two categories of signal shape parameters may only constrain the coarse-grained model parameters to a 1D subset in the {*n*,*q*,*p*} space. In general, to uniquely determine the structure, either an additional independent quantity can be identified by analyzing other shape parameters of *I*(*t*), or alternatively, a constitutive relation among the model parameters or the Fourier transform of *I*(*t*)—known as the empirical characteristic function—can be employed. In the following, we assume for simplicity that the two shape parameters, *log*_10_(*Q*_1/2_) and Δ*Q*_*r*_, suffice to uniquely determine the coarse-grained model parameters.

**Figure 3:**
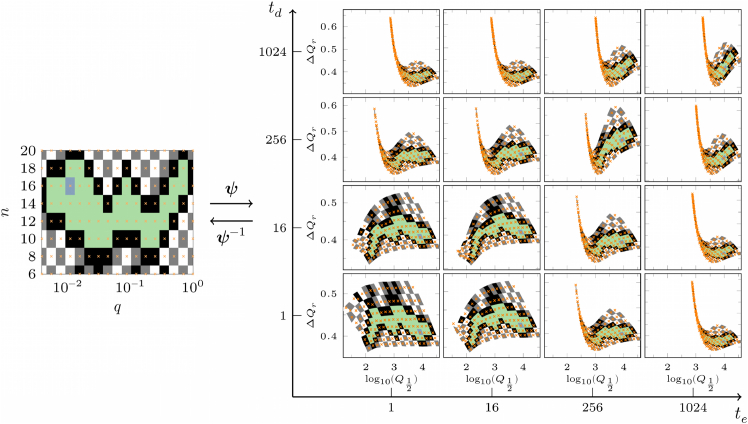
Mapping the structural parameters to the characteristics of the signal intensity (***ψ***) and vice versa (***ψ***^−1^). The mapping of (*q*,*n*) to (*log*_10_(*Q*_1/2_),Δ*Q*_*r*_) (i.e., the logarithm of the median and the relative interquartile range) is presented for different mean entering *t*_*e*_ and emission *t*_*d*_ times. Another parameter value is *p* = 0.55. For every marked point on the structural parameter domain (*yellow crosses*), the corresponding location on the signal characteristic domain is extracted numerically. The neighborhood around each pair of connected points is painted with the same color in both domains for clarity.

**Figure 4:**
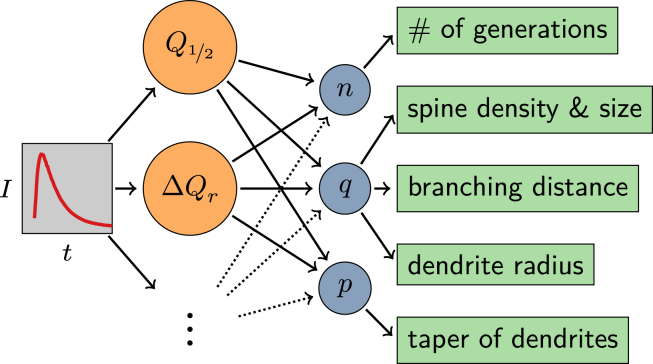
Flowchart of the inference process. Starting from a measured signal (*left*), signal parameters such as the median *Q*_1/2_, the normalized interquartile range Δ*Q*_*r*_, and other possible descriptors (*orange, second column*) are extracted. These parameters are then mapped to the model parameters {*n*,*q*,*p*} (*blue, third column*), which in turn provide information about dendritic morphology (*right*). Arrows indicate the direction of influence among the quantities.

The sensitivity of the relation between the two sets of structural and signal intensity parameters to the choice of *t*_*e*_ and *t*_*d*_ timescales can be more clearly presented in terms of the degree of information compression when mapping the phase spaces of these two sets to each other. We denote the mapping of structure to signal and vice versa with ***ψ*** and ***ψ***^−1^, respectively. Thus, the connection between the two sets can be represented as {*log*_10_(*Q*_1/2_),Δ*Q*_*r*_} = ***ψ***(*n*,*q*,*p*) and {*n*,*q*,*p*} = ***ψ***^−1^(*log*_10_(*Q*_1/2_),Δ*Q*_*r*_) in general. As an example, in Fig. 3, we show the mapping of the (*q*,*n*) plane to the (*log*_10_(*Q*_1/2_),Δ*Q*_*r*_) plane for different mean entering *t*_*e*_ and emission *t*_*d*_ times. We regularly sample the phase space of structural parameters (yellow crosses in left panels) and perform extensive simulations to obtain the signal intensity *I*(*t*) and extract its median *log*_10_(*Q*_1/2_) and dispersion Δ*Q*_*r*_ for each sampled set of {*n*,*q*,*p*}. Fig. 4 summarizes the complete inference process in a flowchart, from extracting statistical features of the signal intensity distribution, to mapping them onto the model parameters, and ultimately inferring dendritic morphological information.

**Figure 5:**
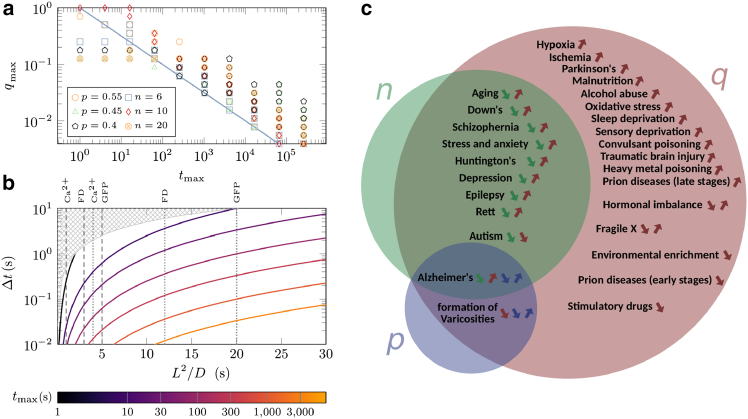
Map invertibility and link between model parameters and pathology of dendrite morphology. (*a*) Invertibility threshold *q*_*max*_ versus the longest timescale *t*_*max*_ = *max*(*t*_*e*_,*t*_*d*_) for different values of *p* and *n*. The line represents $q_{\max}=t_{\max}^{-1/2}$. (*b*) Time resolution of measurements Δ*t* versus diffusive timescale *L*^2^/*D* for different values of *t*_*max*_. The hatched areas indicate the inadmissible region given by Δ*t* >*L*^2^/2*D*, where the probability *q* would be larger than one. The vertical lines mark the relevant range along the *x*-axis for Ca^2+^, fluorescein dextran (FD), and green fluorescent protein (GFP) (59,61). (*c*) Pathologies of dendrite morphology, presented in terms of the mesoscopic model parameters *n*, *p*, and *q* (*green, blue, and red colors, respectively*). Each arrow indicates the increase or decrease of the corresponding parameter in the course of progression of the given disease. See (17,62,63) and references in the main text.

It can be seen from Fig. 3 that for small values of *t*_*e*_ and *t*_*d*_, the structure domain is mapped one to one to the signal domain. The mapping consists of a slightly skewed rotation, but mapping of two or more distinct points of the {*n*,*q*,*p*} parameter space to a same point in the $\left\{\log_{10}\left(Q_{\frac{1}{2}}\right),\Delta Q_{r}\right\}$ space is unlikely (i.e., the map can be inverted). With increasing *t*_*e*_ and *t*_*d*_, the high *q* regions in the structure domain gradually map to highly narrow regions in the signal domain. The compression of information to a space with one less dimension in the limit of large *t*_*e*_ and *t*_*d*_ timescales means that the map cannot be fully inverted anymore. To assess the invertibility limit of *q*, we quantify the compression of the points by the mapping from structure to signal domain; see Supporting Material for details. By setting a threshold level for the information compression, we can determine the maximum value of *q* (denoted by *q*_*max*_) up to which the map remains invertible (Fig. S1). In Fig. 5 *a*, *q*_*max*_ is plotted as a function of *t*_*max*_ = *max*(*t*_*e*_,*t*_*d*_) (i.e., the longest timescale among the entering and emission times). It reveals that the tail of *q*_*max*_ decays with *t*_*max*_ roughly as a power law, which can be presented as $q_{\max}\propto \sqrt{\frac{\Delta t}{t_{\max}}}$ using the fact that *t*_*max*_ is measured in units of Δ*t*. The power law scaling arises only in the asymptotic regime where the entry or emission time dominates the statistics. At low *t*_*max*_, the MFPT dominates the two other timescales, and *q*_*max*_ becomes insensitive to *t*_*max*_. We note that the assessment of the invertibility of the map was conducted empirically based on numerical simulations, and there is currently no rigorous physical derivation for the observed scaling behavior. We also explored possible data collapse in Fig. 5 *a* through rescaling with *n* or *p*. As shown in Fig. S4, by introducing an *n*-dependent prefactor *A*(*n*), a partial collapse of the tails can be obtained across *n* ≤ 20, whereas rescaling with *p* in the biologically relevant range 0.4 ≤ *p* ≤ 0.6 proved ineffective. On the other hand, from Eq. [4] the maximum value of *q* for a given dendritic tree is obtained if spines are absent, leading to $q_{\text{spineless}}=\Delta t\frac{2D}{L^{2}}$, with *D* being the diffusion coefficient in the smooth channel without spines. The full range of *q* is invertible if *q*_spineless_ ≤ *q*_*max*_, which imposes the constraint

(5) $$\Delta t\leq {\left(\frac{L^{2}}{2D}\right)}^{2}\frac{1}{t_{\max}}$$

on Δ*t*, as plotted in Fig. 5 *b* for different values of *t*_*max*_. For a given set of dendritic tree structure and tracer particle, the required time resolution of measurements Δ*t* is inversely proportional to *t*_*max*_. The vertical lines in Fig. 5 *b* mark the relevant range of $\frac{L^{2}}{D}$ for realistic values of the branching distance *L* and diffusion coefficients *D* for Ca^2+^, fluorescein dextran, and GFP, as a few examples. It shows that a slower diffusion of tracer particles and shorter entering and emission times lead to a broader possible range for the time resolution of measurements Δ*t*. For time resolutions around Δ*t* ≃ 0.04 s (typical for currently available cameras) and tracer particles with diffusion coefficients similar to GFP, the required entering and emission times can be up to a few minutes. However, in special techniques such as nuclear magnetic resonance spectroscopy, one deals with time resolutions of several seconds, demanding more slowly diffusing particles and shorter entering and emission times on a subminute scale.

### Morphological changes during disease progression

The morphology of dendrites is broadly affected by aging (19,21,32) or neurodegenerative disorders such as Alzheimer disease (19,20,22,23,24), autism spectrum disorders (19,27,28,29,30), epilepsy disorder (64), schizophrenia (19,25,26), Down syndrome (65), fragile X syndrome (30,31), prion diseases (66), and stress-related disorders (67). The affected morphological properties include the overall extent of dendritic tree, the segmental increase of dendrite diameter toward the soma, the population of branches, the thickness and curvature of dendrite shafts, and the morphology, density, and spatial distribution of spines. Here, we clarify how these morphological changes influence our mesoscopic model parameters {*n*,*q*,*p*}. This information is then used in Fig. 5 *c* to categorize the neurodegenerative disorders—those for which clear trends for the pathological changes of dendrite structure have been reported in the literature—based on the expected trends of the model parameters in the course of disease progression.

Variation of the extent of dendritic tree trivially influences the depth parameter *n*. Reduction of the tree extent is the observed trend during aging and several disorders such as Down syndrome, schizophrenia, Alzheimer disease, autism spectrum disorders, epilepsy disorder, stress-related disorders, Huntington disease, etc. Increasing of the tree extent due to neurodegenerative disorders has not been reported to our knowledge.

The moving probability *q*, given by Eq. (4), is the only parameter affected by the presence of spines. *q* increases with decreasing spine size or density as observed, for example, in aging, Down syndrome, Alzheimer disease, and schizophrenia; see Fig. 5 *c* for an extended list of relevant disorders. Conversely, the spine density increases in a few cases such as autism spectrum disorders, fragile X syndrome, and hormonal imbalance, leading to the decrease of *q*. Nevertheless, the pathology of fragile X makes the prediction of *q* variations complicated: the increase of spine density (decrease of *q*) is accompanied by the shrinkage of spine size (increase of *q*); thus, the two effects compete and may even compensate each other such that *q* remains unchanged. The influence of hormonal imbalance on *q* depends on the hormone type and whether there is a deficiency or surplus.

The disorders that change the width of dendrite shafts influence the bias parameter *p* in general. Particularly, the decrease in dendritic arborization is often correlated with the overall reduction of the channel width. The details of width reduction determine the direction of changes of *p*: although a uniform reduction of channel radii should leave *p* unchanged in the new calibration (and decreases *q*), a radius-dependent reduction may change *p* in both directions. Moreover, an inhomogeneous spatial pattern of *p* can be caused by local changes of the channel width. For instance, local thinning of a channel occurs in the vicinity of amyloid plaque deposition in Alzheimer disease. Finally, the disorders that reduce the population of branches can increase the average node-to-node distance *L*, resulting in smaller *q* and *n*.

We note that the pathology of spine and dendrite structure is more complicated in other diseases. For instance, distortion of spine shape observed in most intellectual disabilities makes the prediction of the trend of *q* difficult. Despite the currently available information discussed above, there is a lack of quantitative studies to clarify the impact of various neurodegenerative disorders on dendritic spine, tree metric, and topological morphology.

## Discussion

Our approach to the search problem on a tree differs from previous approaches, which primarily focus on transport by drift and diffusion (68,69,70,71,72). Those works typically solve the transport equation on individual line segments, impose continuity and flux conservation (Kirchhoff’s law) at junctions, and then solve for concentrations at the junctions in Laplace space, often within a quasi-steady-state approximation of motor-mediated transport (73). By contrast, our coarse-graining is spatiotemporal: rather than resolving concentrations along every dendritic segment, we reduce the dynamics to discrete hopping of tracer particles between bifurcations. This maps the continuous diffusion process in a branched geometry onto a compartmental description, similar in spirit to classical tracer-transport models (74) and more recent treatments of mitochondrial dynamics in branching axons (75). The link to the underlying continuous process is established via first-passage analysis: The discrete model time and real time are related by equating mean number of timesteps to leave a bifurcation with the MFPT for a particle to exit a bifurcation and reach an adjacent one in a continuous three-branch star geometry. The transition probabilities *p* and 1 − *p* for moving to the parent versus child branches are given by the corresponding splitting probabilities.

Since we have been interested in passive diffusion of tracers that explore the dendritic channels and spines in the present work, we retrace the derivation of the Fick-Jacobs equation for diffusion in a channel branching into identical child channels, yielding a 1D Smoluchowski-type equation for transport of tracers in a three-pointed star geometry (note that the 1D description remains more generally valid for coordination numbers *z* > 3). This guarantees conservation of current at the bifurcations and allows analytical evaluation of MFPTs and splitting probabilities. Importantly, such closed-form results are generally not available when solving for full concentration profiles with spatially varying drift or diffusion coefficients. Although our derivation here focuses on passive diffusion, the framework is readily extendable to include drift terms, thereby connecting back to quasi-steady-state approximations of motor-driven transport (73). A limitation of our coarse-grained model is that it naturally restricts target locations to bifurcations, whereas PDE-based approaches can also describe targets located along the branches (68).

We have demonstrated that the parameters {*n*,*q*,*p*} of our mesoscopic model can be extracted by analyzing the detectable temporarily signal generated by a large population of neurons, provided that the timescales of entering the dendrites and emission of signal after reaching the soma are sufficiently small compared with the MFPT of passing the dendritic tree. Although we constrained our analysis to signals formed by spontaneous pulses emitted by the particles in the soma with activation probability $\frac{1}{t_{d}}$ (*t*_*d*_ is measured in units of Δ*t*, i.e., the resolution of measurement or observation), signals of other forms can be easily obtained from the signal studied in the present work. For example, if one seeks insight into the ability of neurons to integrate spine-derived (concentration) signals, the number of particles in the soma that have not yet emitted their pulse (i.e., are still active in this case) would be of interest. This quantity at time *t* is given by *t*_*d*_*I*(*t* + 1) (i.e., our measured signal shifted by one time step to the left and scaled by the mean activation time). In Fig. S3, we present this signal alongside the time evolution of the fraction of particles in the soma that have not yet emitted their pulse for different values of *t*_*e*_ and *t*_*d*_ and for healthy versus differently degenerated dendritic trees.

On the other hand, the model parameters {*n*,*q*,*p*} can be directly linked to the morphology of real dendrites via Eqs. (3) and (4). Since there are several morphological characteristics on the right-hand sides of these equations, they cannot be uniquely determined by a given set of {*n*,*q*,*p*}. Nevertheless, most neurodegenerative disorders affect only a few of the morphological properties of dendrites. Therefore, by conducting regular patient monitoring for a given disease, the observed changes in the parameters {*n*,*q*,*p*} can be attributed to the changes of the morphological properties relevant to that specific disease. For instance, the growth rate of *q* and reduction rate of *n* for a patient with schizophrenia reflect, respectively, how fast the mean spine volume and the extent of dendritic tree are shrinking over time.

To link the detected signal intensity to the mesoscopic model parameters, we have considered an ideal regular tree structure, whereas real dendritic trees are irregularly branched, spines have diverse sizes, and their spatial distribution is inhomogeneous. These fluctuations naturally cause variations in the corresponding model parameters {*n*,*q*,*p*}. However, we verified in our previous study (45) that the analytical results for the first-passage times of passing a regular tree structure remain valid when realistic degrees of global fluctuations of the structural parameters across the tree or local structural irregularities in the branching patterns are considered. Since the dependence of the signal intensity on the dendrite morphology is due to the contribution of the fist-passage times (and not the entering *t*_*e*_ and emission *t*_*d*_ times), we conclude that the presented results in the current study remain valid under typical structural irregularities and fluctuations observed in real neuronal dendrites.

We have characterized the behavior of the signal intensity *I*(*t*) by two quantities, the logarithm of the median *log*_10_(*Q*_1/2_) and the relative interquartile range Δ*Q*_*r*_. The former is a representative of a category of the statistical measures including the mean, median, and maximum of *I*(*t*). The latter quantifies the statistical dispersion of *I*(*t*) and behaves similar to quantities such as the normalized variance and skewness. One may still identify further independent quantities by analyzing other moments of *I*(*t*). Additional statistical measures of *I*(*t*)—which vary smoothly with the parameters {*n*,*q*,*p*} and exhibit isosurfaces that differ from those of *log*_10_(*Q*_1/2_) and Δ*Q*_*r*_—can in principle improve the accuracy of the extracted values of the model parameters {*n*,*q*,*p*}.

**Figure 6:**
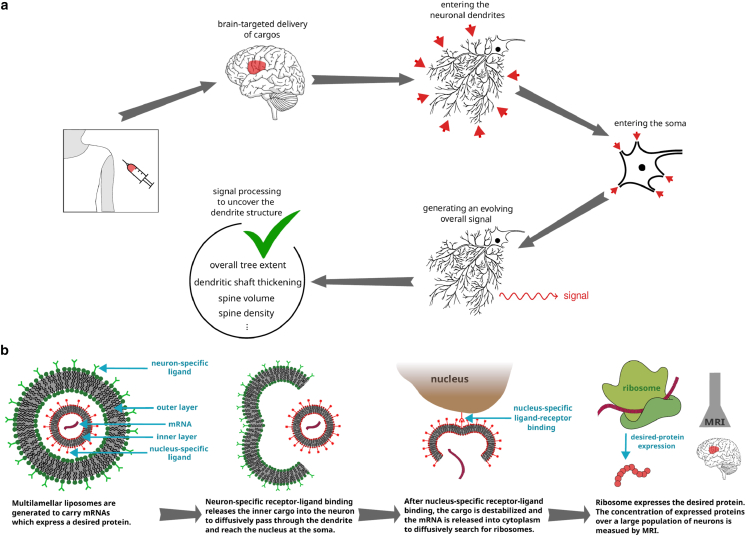
Schematics of our proposed approach and a possible design for generating an externally detectable signal. (*a*) Schematic of our proposed noninvasive technique for a fast indirect measurement of the structural properties of dendrites based on processing a signal generated by a large population of neurons. By conducting regular measurements for a given patient, essential information about the morphological evolution of dendrites in the course of neurodegenerative disease or treatment progression can be extracted. (*b*) Schematic illustration of multilamellar liposomes designed to generate a specific protein. As a detectable temporary signal, the concentration of the expressed protein across the brain region of interest is externally monitored by the MRI technique.

Our work primarily addresses the theoretical and computational problem of linking the statistical properties of a measured signal to the underlying dendritic morphology through a stochastic coarse-grained first-passage framework. The present study does not aim to design or implement an experimental setup for generating such a signal. Nevertheless, to illustrate the potential applicability of the framework and to motivate future experimental efforts, we briefly outline possible routes toward generating a detectable signal. This involves the transport of tracer or cargo molecules to selected brain regions, their entry into dendrites, subsequent traveling toward the soma, and the production of a measurable transient signal. The conceptual workflow of this process is summarized in Fig. 6 *a*. Although addressing the technical challenges associated with these steps lies beyond the scope of this work, we note that achieving a detectable signal appears feasible with currently available technologies. Below, we discuss potential strategies for each stage of this illustrative measurement procedure.

1. **Transport of cargos to desired regions of brain tissue by means of brain-targeted drug delivery techniques.** Promising strategies have been developed so far to deliver drugs specifically to the brain to treat neurological disorders while minimizing systemic side effects (76,77,78). Some of the currently feasible techniques include nanoparticle-based delivery (76,79,80,81,82,83,84,85,86), focused ultrasound (noninvasive technique that offers spatially targeted drug delivery by transiently disrupting the blood-brain barrier (BBB) to pass through) (87), and carrier-mediated transport (utilizing endogenous transport systems like glucose or amino acid transporters facilitates drug transport across the BBB; drug molecules are conjugated with ligands that target these transporters to enhance brain uptake) (88). Moreover, injection into the spinal cord fluid or into ventricles could be an option to pass the BBB as well.
2. **Entering the neuronal dendrites and passing through their complex structure to reach the soma.** After reaching the area around the neurons, the contents of the cargos can enter the neuron by means of neuron-specific receptor-ligand binding (81,82,83,84,85,86). This would mainly occur through the dendritic tree rather than axon or soma since the outer area of the neuron is mainly formed by the dendritic tree. Nevertheless, the contribution of entering from soma or axon to the generated signal can be evaluated and subtracted, as long as the axons and somata do not undergo morphological changes in the course of disease progression or treatment.
3. **Generating a temporary signal and detecting it.** Here, we mean any kind of detectable signal such as, but not limited to, electric or magnetic fields generated by many neurons. There are powerful noninvasive techniques for real-time tracking of brain activities. Electro- and magneto-encephalography for electric and magnetic field detection are established neurotherapeutic tools (39,40). Another possibility is to employ nuclear magnetic resonance spectroscopy, which allows for noninvasive measurements of the concentration of different neuro-chemicals within a volume of brain down to a few cubic centimeters (41,42). The concentrations of substances generated in the somata of neurons can be obtained via nuclear magnetic resonance spectroscopy with a time resolution of a few seconds, which, depending on the diffusion constant of the particles, can remain within the feasibility range of our proposed method (89). Positron emission tomography and MRI can be also employed to measure the concentration of neuro-chemicals (43,44).
4. **Processing the evolving overall signal.** After detecting the signal, the approach developed in this paper enables processing the signal intensity to uncover dendritic morphologies.

As a more detailed plan for generating a detectable signal, we propose a protein expression scenario by injecting specific mRNAs carried by multilamellar liposomes; see Fig. 6 *b*. The concept of producing multilamellar liposomes is currently feasible and has been realized in the context of cell activity regulation, immunotherapy, and vaccination (90,91,92). Transport of liposomes to desired regions of brain and uptake of them by neurons have been feasible by modifying their surface with ligands targeting specific receptors on brain endothelial cells or neuronal dendrites (81,82,83,84,85,86). Upon neuron-specific receptor-ligand binding, the multilamellar liposome enters the dendrite and loses its outer layer, leading to the release of the inner cargo into the cytoplasm. To enhance the dendrite-specific entering, the endocytosis events around the synapses can be harnessed. For example, there is evidence that AMPA receptors are preferentially endocytosed around synapses (93). The inner cargo is conjugated with nucleus-specific ligands (94,95,96) and diffuses inside the dendritic tree until it enters the soma and reaches the nucleus. The cargo can be designed to be destabilized or dissolved after the nucleus-specific receptor-ligand binding pins it to the exterior of the nucleus. This can be achieved, for example, through specific proteolytic enzymes or pH-sensitive components in the cargo structure or adjusting the concentration of aqueous ionic solutions inside the cargo (97) to respond to the environmental differences between the region around the nucleus and the rest of the cytoplasm. The destabilized cargo releases mRNAs into the cytoplasm, which will diffusively search for ribosomes to produce a desired protein, such as ferritin. A typical neuron contains millions of ribosomes, but their homeostatic distribution is still unknown (98,99) (though recent studies revealed spatial inhomogeneities in the protein translation across the neuron, attributed to the spatial distribution of mRNAs and potential local specialization of ribosomes (99,100)). The expressed protein should be harmless and degrade over a reasonable time. Variations of the concentration of this protein over a large population of cells can be externally detected. For example, expression of ferritin can be monitored by the MRI technique (44). We note that the presented formalism in the previous sections to obtain the first-passage time distribution of reaching the soma can be straightforwardly extended to calculate the additional first-passage time distribution of reaching from the soma to the distributed ribosomes. Moreover, here, we considered a spontaneous signal emission, but the formalism can be adapted to other scenarios such as a gradually degrading signal.

The clinical translation of these proposed techniques certainly requires rigorous testing and validation to ensure the safety and specificity of the novel approaches. Uptake and transport of cargos can be initially tested, for example, in cultured murine neurons. For the plan proposed in Fig. 6 *b* based on the expression of proteins by ribosomes, spatial distribution of ribosomes in different types of healthy neurons needs to be determined. We expect that our proposals can potentially trigger active research and development in the fields of neuroscience, molecular engineering, and pharmacology.

To conclude, a framework has been developed to link the statistical characteristics of a detectable signal generated after reaching the somata of neurons to the morphological properties of neuronal dendrite structures. Our results open the possibility of indirectly monitoring the morphological evolution of dendrites in the course of neurodegenerative disorder progression. The mesoscopic approach presented in this study can be generalized to cope with further details of transport in real dendrite structures, such as handling the memory effects and aging inside spines (101) or to include active transport of cargos along microtubules (102). Besides drawing conclusions regarding the morphological changes of dendrite structures, investigation of the first-passage properties of stochastic motion inside dendrites can deliver vital information about the ability to preserve local concentrations or induce concentration gradients of ions and molecules. These are tightly connected to neural functions and allow for drawing important physiological conclusions. The proposed approach also provides a route into a variety of other stochastic transport phenomena (e.g., in varying energy landscapes, branched macromolecules and polymers, and labyrinthine environments with absorbing boundaries).

## Acknowledgments

We would like to thank Jochen Hub and Chetan Poojari for fruitful discussions and Anne Hafner for helping us with the schematic drawing of a neuron in Fig. 1. We acknowledge the use of GNU Parallel for scheduling and running our simulations in parallel (103). This work was supported by the 10.13039/501100001659Deutsche Forschungsgemeinschaft (10.13039/501100001659DFG) within the collaborative research center SFB 1027 and also via grants INST 256/539-1, which funded the computing resources at Saarland University. R.S. acknowledges support by the Young Investigator Grant of Saarland University, grant no. 7410110401.

## Author contributions

R.S. designed research; F.H.K., L.S., and R.S. developed the model; F.H.K. performed simulations; all authors contributed to the analysis and interpretation of the results; F.H.K. and R.S. wrote and all authors revised the paper.

## Declaration of interests

The authors declare no competing interests.

## Supporting material

Document S1. Figures S1–S4

Document S2. Article plus supporting material
