# Supplementary material for: Tracking the morphological evolution of neuronal dendrites by first-passage analysis
Source: Biophys J. 2025 Nov 7;125(1):64–76. doi: 10.1016/j.bpj.2025.11.005 (PMC12821030; doi:10.1016/j.bpj.2025.11.005)
Supplement: Document S1. Figures S1–S4 [file mmc1.pdf]

Biophysical Journal, Volume *125*

Supplemental information

Tracking the morphological evolution of neuronal dendrites by first-passage analysis

Fabian H. Kreten, Barbara A. Niemeyer, Ludger Santen, and Reza Shaebani

Supplementary Information to Tracking the Morphological Evolution of Neuronal Dendrites by First-Passage Analysis

Fabian H. Kreten, Barbara A. Niemeyer, Ludger Santen, Reza Shaebani*

*To whom correspondence should be addressed: shaebani@lusi.uni-sb.de

Mapping dendritic structure to the model parameter q

The mean escape time $\langle t\rangle$ from a junction to any neighboring furcation can be expressed, on the one hand, in terms of channel geometry as $\langle t\rangle = \frac{L^2}{2D}$, assuming diffusive dynamics. On the other hand, using the discrete-time framework of the model with observation time resolution Δt, it is given by $\langle t\rangle = \frac{\Delta t}{q}$. Equating these two expressions yields a relation between the moving probability q and the geometric parameters of the dendritic structure, $q = \Delta t\, \frac{2D}{L^2}$. However, we have assumed a smooth channel so far, thus, this relation does not yet account for the effects of trapping in dendritic spines. In the following, we show how these effects can be incorporated into the framework.

We note that transient trapping events along the channel do not induce any bias in the motion towards one end of the channel segment, thus, no modification is required in the calibration relation for the parameter p. However, for the escape time $\langle t\rangle$, frequent interruption of motion by entrapment events in spines has a considerable impact. To keep the model traceable, this impact is taken into account by an effective asymptotic diffusion constant D_{eff}. Previous studies have already calculated such an effective diffusion constant in a geometry almost tailored to the diffusive transport in spiny dendrites [1]. The geometry considered there consists of a cylindrical tube from which identical spines protrude periodically. The spines were modeled as spherical cavities connected to the main shaft by narrow cylindrical necks. The effective diffusion constant derived in [1], adapted to our application, is given by

$$D_{\text{eff}} = D\frac{V_{\text{channel}}}{V_{\text{channel}} + V_{\text{spines}}} = D\frac{1}{1+\frac{V_{\text{spines}}}{V_{\text{channel}}}}, \tag{1}$$

where D is the diffusion constant without protrusions, V_{channel} the channel volume, and V_{spines} the total volume of spines. Let us assume a uniform distribution of spines along the dendritic channel with the density ρ per length unit. Denoting the spine head volume with V_{head} and neck volume with V_{neck}, the ratio $\frac{V_{\text{spines}}}{V_{\text{channel}}}$ can be written as

$$\frac{V_{\text{spines}}}{V_{\text{channel}}} = \frac{\rho(V_{\text{head}} + V_{\text{neck}})}{\pi R^2}, \tag{2}$$

where R is the channel radius. Substituting D_{eff} into the calibration relation for q yields

$$q = \Delta t\frac{2D}{L^2}\frac{V_{\text{channel}}}{V_{\text{channel}} + V_{\text{spines}}} = \Delta t\frac{2D}{L^2}\frac{1}{1+\frac{\rho(V_{\text{head}}+V_{\text{neck}})}{\pi R^2}}. \tag{3}$$

As q is a probability, it cannot be larger than one, imposing a constraint on the time resolution of observation Δt. Since the relation $\frac{V_{\text{channel}}}{V_{\text{channel}}+V_{\text{spines}}} \leq 1$ always holds, the condition

$$\Delta t \leq \frac{L^2}{2D} \tag{4}$$

ensures that q always remains as a valid probability, i.e., $q \leq 1$.

To derive the above calibration relation, we have made a few simplifying assumptions for the diffusive dynamics of tracer particles inside dendritic channels. For example, a constant distance L between successive junctions is assumed. However, L may vary in real dendrite structures, not only between the segments within one generation but also between different generations. The primary segments of apical dendrites of pyramidal

neurons and terminal segments of all dendrites are reported to be longer than intermediate segments. As a result, q should slightly vary with the depth of dendritic tree due to its L-dependence.

Additionally, spines are inhomogeneously distributed over the dendritic tree. There are almost no spines very close to the soma but the spine number density rapidly grows and saturates after a short distance from the soma. However, the gradual thinning of the channel towards dendritic terminals practically increases the trapping probability inside spines and, hence, decreases q.

Nevertheless, we verified in our previous study [2] that the analytical results for the first-passage times of passing a regular tree structure remain valid when realistic degrees of global fluctuations of the structural parameters across the tree or local structural irregularities in the local branching patterns are considered. To conclude, the structural parameters which enter into the calibration relations for q and p parameters should be considered as average values over the entire dendritic tree.

Considerations for choosing the time resolution of measurements

In this section we discuss the choice of the time resolution of measurements Δt and provide a rough estimate of the applicability range of our proposed method. Figure 3 of the manuscript revealed that the invertibility of mapping the structural parameters to the signal characteristics breaks down for large entering t_e and/or emission t_d times. We further clarified that only the mapping of high q regions is problematic, while low q regions can be resolved even for very large values of t_e and t_d. To assess the invertibility limit of q, in the following we quantify the compression of the points by the mapping. For any given point in the structural parameter space and the corresponding point in the signal domain, the degree of compression is determined by calculating two distances: the minimum distance ℓ_{struct} between the selected point and all other points in the structural parameter space and the minimum distance ℓ_{signal} between the corresponding point and all other points in the $\big(\log_{10}(Q_{\frac{1}{2}}), \Delta Q_r\big)$ plane of signal characteristics. In each of the two domains, the distance between a pair of points (x_1, y_1) and (x_2, y_2) is calculated using the metric

$$\delta\big(\big(\begin{smallmatrix} x_1 \\ y_1 \end{smallmatrix}\big), \big(\begin{smallmatrix} x_2 \\ y_2 \end{smallmatrix}\big)\big) = \sqrt{\left(\frac{x_2 - x_1}{u_x}\right)^2 + \left(\frac{y_2 - y_1}{u_y}\right)^2}, \tag{5}$$

where (x_i, y_i) can be any pair of the structural parameters $\{p, q, n\}$ or a point in the $\big(\log_{10}(Q_{\frac{1}{2}}), \Delta Q_r\big)$ plane of signal characteristics. u_x and u_y denote the total variation range along x and y axes, respectively. Note that u_x and u_y in the signal domain are determined by combining the variation range of $\log_{10}(Q_{\frac{1}{2}})$ or ΔQ_r over all choices of the entering and emission times.

From the minimum distances ℓ_{struct} and ℓ_{signal}, the volumes of the neighbourhoods in the two domains can be estimated as ℓ^2_{struct} and ℓ^2_{signal}, respectively. We introduce the ratio of the volumes $C = \frac{\ell^2_{\text{signal}}}{\ell^2_{\text{struct}}}$ (hereafter refereed to as compression ratio) as a measure of the degree of mapping compression. An invertible mapping requires $C > 0$. In this regard, the compression ratio has similarities with the Jacobian determinant of the mapping. The difference between them is that the distances to all other points in each domain are taken into account in C whereas for the Jacobian only the neighborhood in each domain enters. Therefore, C is a stronger measure for invertibility because it vanishes even when a point far away from the one where C is calculated is mapped to the same point in the signal domain, but the Jacobian determinant cannot capture it due to its local nature. In the case of a globally invertible map, C is an estimate for the absolute value of the Jacobian determinant.

Setting a lower compression threshold $C_{\min}$ allows us to identify the points for which the invertibility of the map between structural and signal domains practically breaks down. By choosing a threshold value $C_{\min} = 0.05$, we identify the points where mapping the structural parameters to the signal characteristics plane is not invertible. This procedure is visualized in Fig. S1 for a constant p and various entering and emission times. Next, we determine $q_{\max}$ as the maximum value of q up to which the map is invertible for all values of the other dimension of structural parameters (n in the cases presented in Fig. S1). We checked that the choice of the threshold level $C_{\min}$ has no qualitative impact on the behavior of $q_{\max}$ and only induces minor quantitative changes.

In Fig. S2, $q_{\max}$ is plotted as a function of the entering time t_e and the emission time t_d for a given value of p. It can be seen that the isolines of constant $q_{\max}$ are roughly square-shaped which evidences that $q_{\max}$ is a function of the largest time scale, i.e. $t_{\max} = \max(t_e, t_d)$. This is confirmed in Fig. 4(a) of the manuscript, where $q_{\max}$ is plotted as a function of $t_{\max}$ for different values of the structural parameters. The overall trend of $q_{\max}$ can be roughly captured by a power-law scaling $q_{\max} = 1/\sqrt{t_{\max}}$. The observed deviations from the power-law scaling originate from the intrinsic stochasticities as well as our method of compression ratio calculation. For small values of t_e, t_d and p and large values of n, the points of the structural parameters domain are mapped onto a patch with low but nonzero extension along the ΔQ_r direction in the signal domain. By increasing the entering or emission time, the points collapse on a nonmonotonic curve which covers a much larger range along ΔQ_r direction; see Fig. S1(b). This results in larger u_x or u_y in the metric Eq. [5], thus, smaller C values. Hence, those points may be considered as non-invertible despite that their mapping to the signal domain is properly resolved.

From the power-law relation between $q_{\max}$ and $t_{\max}$ and the fact that the time scales t_e and t_d are measured in units of the time resolution of observation Δt, it reads

$$q_{\max} = \sqrt{\frac{\Delta t}{t_{\max}}}. \tag{6}$$

According to Eq. [3], the value of q for a given dendritic tree can be almost arbitrarily tuned through Δt. If the upper estimate of q from Eq. [3] (obtained for a smooth channel, i.e. V_{spines}=0) is less than $q_{\max}$ given by Eq. [6], variations of q due to morphological changes of spines can be fully resolved with our proposed approach. By equating Eq. [6] with Eq. [3] at V_{spines}=0 we obtain the relation

$$t_{\max} = \frac{1}{4\Delta t}\left(\frac{L^2}{D}\right)^2 \tag{7}$$

between $t_{\max} = \max(t_e, t_d)$, the time resolution Δt, and the diffusive timescale $\frac{L^2}{D}$ (D is the diffusion coefficient of the tracer particles in the spineless dendritic channel). Therefore, for a given set of dendritic tree structure and tracer particle, the required time resolution of measurements Δt is inversely proportional to $t_{\max}$, i.e. the maximum time scale among the entering and emission times t_e and t_d. In Fig. 4(b) of the manuscript, Δt is plotted versus the diffusive timescale $\frac{L^2}{D}$ for different values of $t_{\max}$. The vertical lines mark the relevant range of $\frac{L^2}{D}$ for realistic values of the branching distance L and diffusion coefficients D for Ca^{2+}, fluorescein dextran (FD), and green fluorescent protein (GFP), as a few examples. It shows that a slower diffusion of tracer particles and shorter entering and emission times lead to a broader possible range for the time resolution of measurements Δt. For time resolutions around $\Delta t \simeq 0.04$ s (typical for currently available cameras) and tracer particles with diffusion coefficients similar to GFP, the required entering and emission times can be up to a few minutes. However, in special techniques such as nuclear magnetic resonance spectroscopy, one deals with time resolutions of several seconds, demanding more slowly diffusing particles and shorter entering and emission times on a sub-minute scale.

[1] L. Dagdug, A. M. Berezhkovskii, Y. A. Makhnovskii, and V. Y. Zitserman, J. Chem. Phys. **127**, 224712 (2007).
[2] R. Jose, L. Santen, and M. R. Shaebani, Biophys. J. **115**, 2014 (2018).

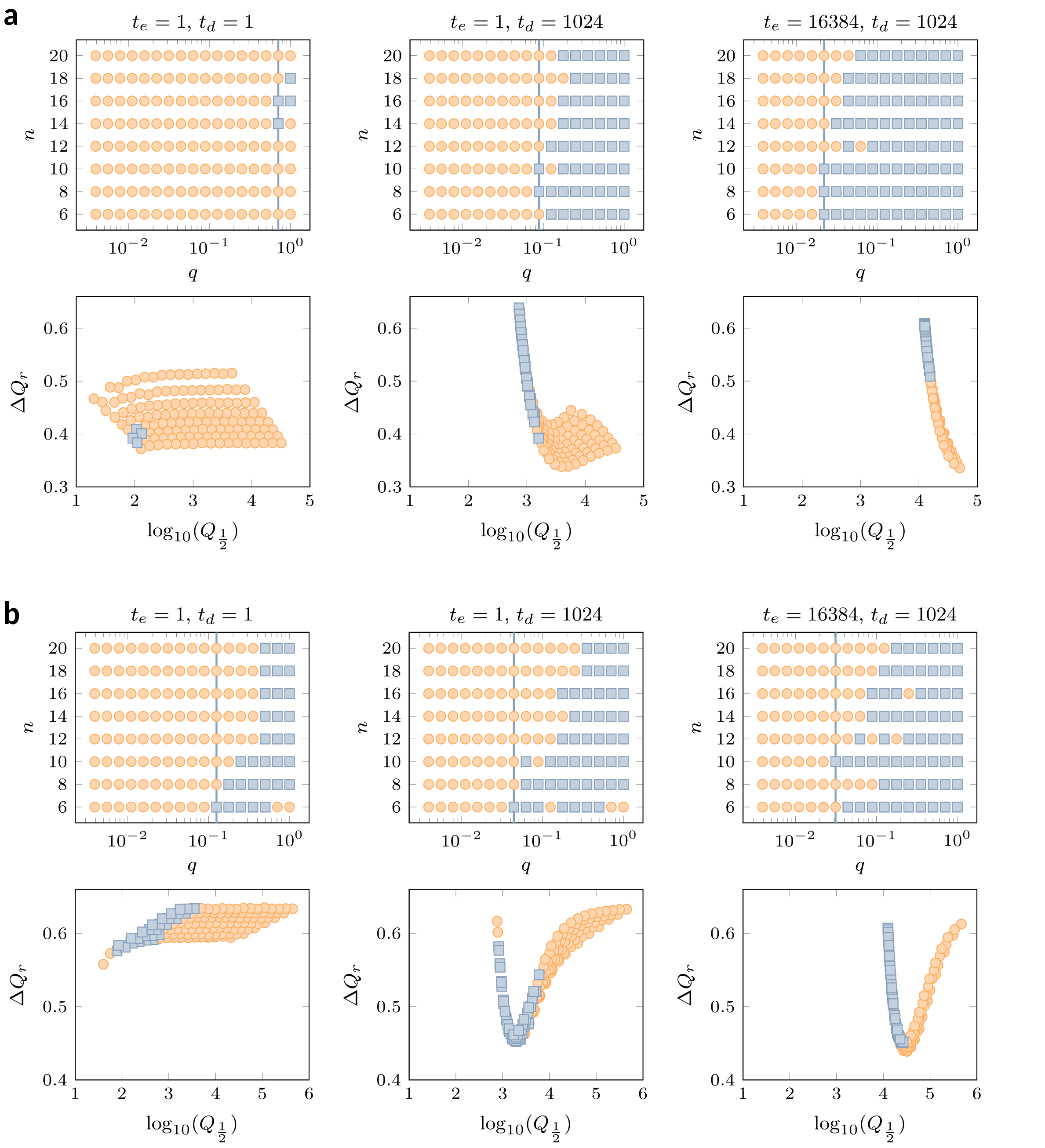

Suppl. Fig. S1: Visualisation of mapping the structural parameters (q, n) to the signal characteristics $\left(\log_{10}(Q_{\frac{1}{2}}), \Delta Q_r\right)$ for different entering times t_e and emission times t_d. Other parameter values: (a) $p = 0.55$, (b) $p = 0.45$. The points where the map is invertible (not invertible) for the compression threshold $C = 0.05$ are shown with orange circles (blue squares). The blue vertical line marks $q_{\max}$, up to which the map is invertible for all values of n.

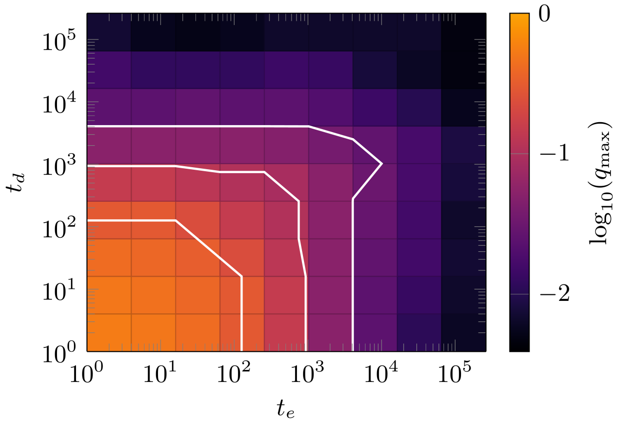

Suppl. Fig. S2: Logarithm of $q_{\max}$ (i.e. the maximum value of q up to which the map from (q, n) to signal domain is invertible) as a function of entering and emission times t_e and t_d. $q_{\max}$ is extracted for $p = 0.55$ and $C_{\min} = 0.05$. The solid white lines represent isolines of constant $q_{\max}$.

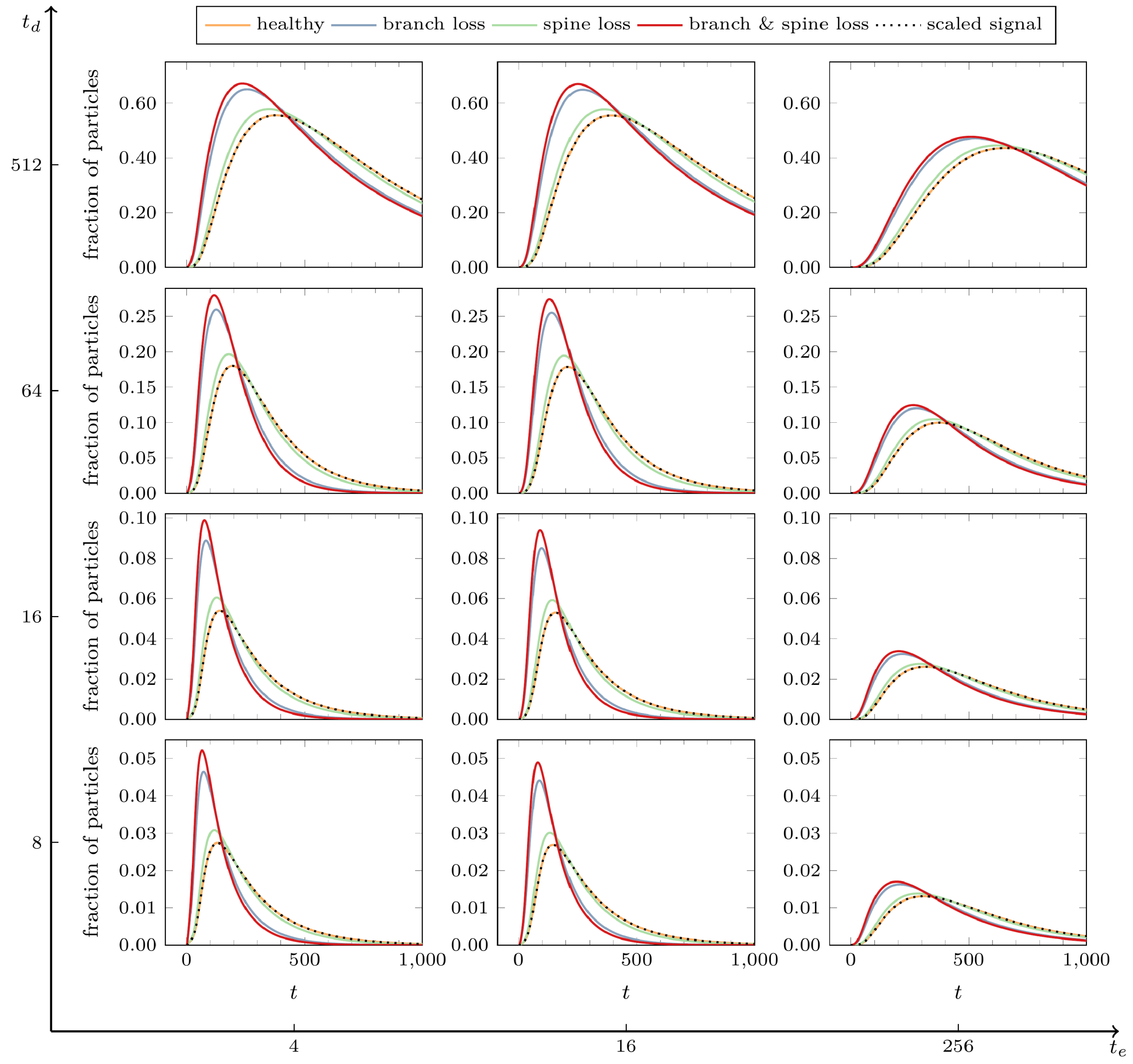

Suppl. Fig. S3: Fraction of particles in the soma as a function of time for healthy and differently degenerated dendritic trees for different entering and emission times t_e and t_d. For the healthy dendrite (orange line) the following parameters were assumed: $n = 10$, $\rho = 1\,\mu\text{m}^{-1}$, $V_{\text{head}} + V_{\text{neck}} = 0.55\,\mu\text{m}^3$ and $R = 1\,\mu\text{m}$. The time resolution was chosen to be $\Delta t = \frac{L^2}{8D}$ corresponding to $\Delta t = 2.5\,\text{s}$ for a dendrite with mean branch length $L = 20\,\mu\text{m}$ and particles with a diffusion constant D similar to GFP. The degeneracies were branch loss (blue line) where the tree has lost three generations of branches, spine loss (green line) where the dendrites have lost three quarters of their spine volume as well as the combination of both (red line). Increasing t_d increases the fraction of particles in the soma leading to a broader and higher curve. Increasing t_e, on the other hand, leads to broader and flatter curves because of the restricted influx of particles. For the healthy dendrite, the signal $I/\mathcal{N}$ (generated by the accumulated pulses of the particles in the soma scaled by t_d and shifted by one step to the left) is shown with the dotted line. This line coincides with the one for particle fraction, exemplifying that the particle fraction in the soma can be obtained by the signal and vice versa.

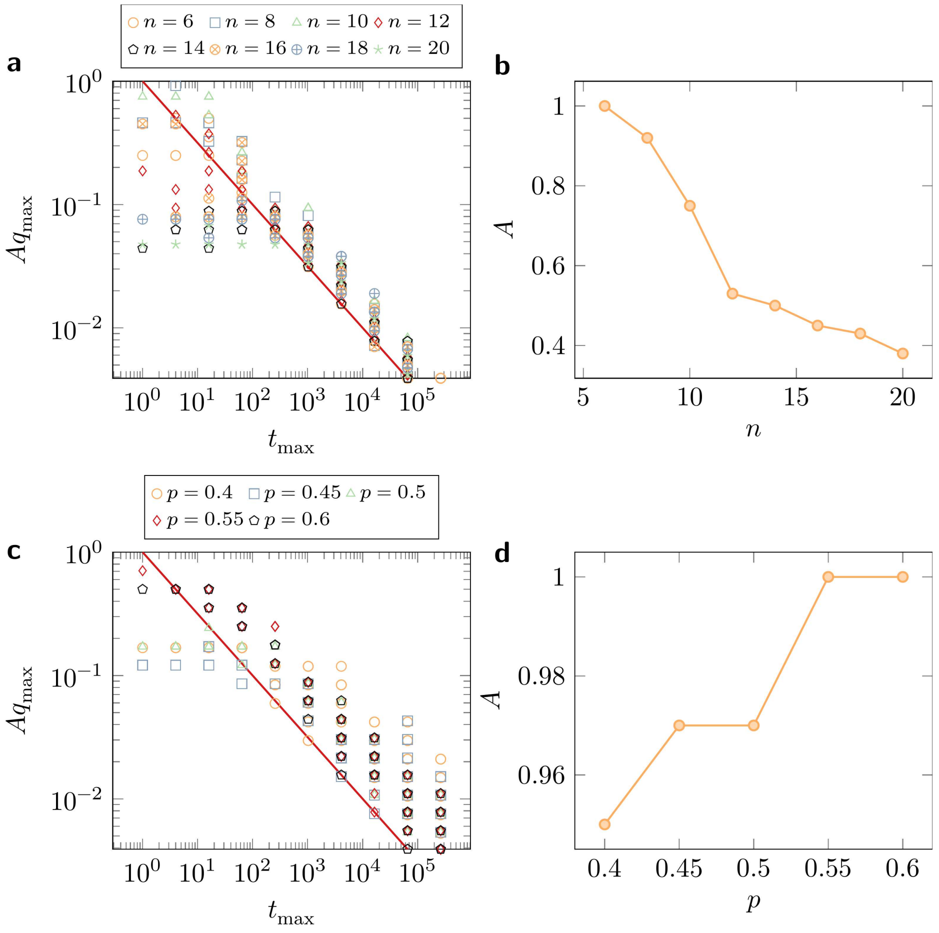

Suppl. Fig. S4: (a) Invertibility threshold $q_{\max}$, rescaled by an n-dependent prefactor $A(n)$, versus the longest time scale $t_{\max} = \max(t_e, t_d)$ for different values of n. The line represents $q_{\max} = t_{\max}^{-1/2}$. (b) The rescaled value $A(n)$ versus n. (c) $q_{\max}$, rescaled by a p-dependent prefactor $A(p)$, versus $t_{\max}$ for different values of p. (d) $A(p)$ versus p. While a partial collapse of the tails across $n \leq 20$ is obtained in panel (a), rescaling with p in the biologically relevant range $0.4 \leq p \leq 0.6$ proved ineffective in panel (c), possibly due to the narrow variation of p or the inherent spread of $q_{\max}$ values. Since rescaling primarily shifts curves to higher $q_{\max}$ without altering the slope, the inferred upper bound for time resolution remains valid.
